# Early onset retinal dystrophies: clinical clues to diagnosis for pediatricians

**DOI:** 10.1186/s13052-019-0760-5

**Published:** 2019-12-21

**Authors:** Agnese Suppiej, Silvia Marino, Maria Eleonora Reffo, Veronica Maritan, Giovanna Vitaliti, Janette Mailo, Raffaele Falsaperla

**Affiliations:** 1 Department of Medical Sciences - Pediatric Section, University of Ferrara, Ferrara, Italy; 2 Robert Hollman Foundation, Padua, Italy; 3 Paediatric Operative Unit and Acute and Emergency, Policlinico Vittorio Emanuele University Hospital of Catania, Catania, Italy; 4 Ophthalmology Unit, Azienda ULS 6, Euganea, Italy; 5 Division of Pediatric Neurology, Stoller Children’s Hospital, Edmonton, Alberta Canada

**Keywords:** Blindness, Child, Low vision, Electroretinography, Nystagmus

## Abstract

**Introduction:**

Inherited retinal dystrophies are major cause of severe progressive vision loss in children. Early recognition and diagnosis are essential for timely visual rehabilitation during the appropriate stages of the visual development, as well as for genetic diagnosis and possible gene therapy. The aim of this study is to characterize a pattern of the initial visual symptoms, which could help the pediatricians and the primary care providers to suspect an inherited retinal disorder in its early stage.

**Methods:**

We analyzed the initial clinical symptoms, based on parental report during the first visit to specialist, in 50 children diagnosed with retinal dystrophy confirmed by full-field electroretinography. The analysis included the age of symptoms onset and the type of visual symptoms, both in the total population and in the following diagnostic subgroups: rod-cone dystrophy (n.17), cone-rod dystrophy (n.12), achromatopsia (n.13), congenital stationary night blindness (n.6) and Leber’s congenital amaurosis (n.2).

**Results:**

The majority of children (80%) had the onset of clinical symptoms before one year of age. The most frequent visual complaints reported by parents were nystagmus (76%), visual loss (28%) and photophobia (8%). Nystagmus was the first symptom reported by parents if the disease onset was before the age of six months, while the onset after the six months of age was more likely associated with the complain of vision loss.

**Conclusions:**

Low vision and nystagmus observed by parents, particularly in the first year of life, may represent a red flag, prompting an appropriate ophthalmological workup for inherited retinal dystrophy.

## Introduction

Inherited Retinal Dystrophies (IRDs) are a major cause of severe progressive vision loss in children. They include a large group of clinically and genetically heterogeneous disorders that affect approximately 1 in 3000 patients [1], with more than 271 causative genes identified to date (RetNet: https://sph.uth.edu/retnet/ 9 March 2019).

At early stages, the fundus oculi examination may be normal or not specific. Furthermore; the diagnostic tests that can reveal features of IRDs in adults [2] are often either difficult to perform or not reliable in very young children. Research shows that the electroretinogram (ERG) can be used as an objective assessment method, independent of the child’s cooperation. Its diagnostic role has been now well established in pediatric IRDs [3–6]. The ERG evaluates the retina-wide function of rods and cones, and it can confirm a clinical suspicion for diffuse photoreceptor disease, the most frequent IRD types in childhood [7]. However, in the absence of clear clinical clues, clinicians are often reluctant to prescribe ERG testing in young children, potentially prolonging the time to diagnosis.

In adult IRD patients, the retrospective evaluation of the initial symptoms pattern was proven useful in providing additional clues for early disease recognition and also for inclusion in clinical trials [7]. Similar studies are not available in the pediatric population. The initial manifestation of IRD depends on the specific type of photoreceptor dysfunction. Color blindness, low vision, increased sensitivity to bright lights and nystagmus represent cones dysfunction [8, 9]; while night vision problems and reduced peripheral visual fields are associate with rods dysfunction [10]. In the progressive forms of IRDs, there can be an initial dysfunction of the cones, followed by a progression to rods dysfunction, or alternatively, the IRD can affect rods first and then progress to cones [11–13].

We believe that the identification of clinical clues that may suggest early IRDs in children may improve the time to diagnosis. Early diagnosis is important for the access to visual rehabilitation during the appropriate developmental window, for further investigations, but increasingly also for a genetic diagnosis, and parental genetic counselling. Indeed, timely recognition and optimal multidisciplinary management of these disorders can have very important implications for improving children’s quality of life [4]. Furthermore, gene replacement therapies are on the way for some IRDs and in those cases the early diagnosis will be essential [14–16].

The aim of this study was to analyze the initial signs and symptoms of IRD as reported by the parents at the initial ophthalmological visit, in a cohort of 50 children diagnosed with IRD. The ultimate goal was to identify clinical clues that will help the pediatrician to suspect a retinal dystrophy.

## Subjects and methods

The study group included 50 patients (30 males), diagnosed with isolated retinal dystrophy, and confirmed by the ERG. Children were all born at term, except one born at 36 weeks gestation. Mean gestational age was 39 weeks (range 36–42). Mean age 4 years and eight months (range 5 months − 14 years), The patients were recruited from the database of our institutions. The patients’ data were prospectively collected over a period of 7 years, following an initial clinical evaluation by a paediatric ophthalmologist and electroretinography. Children were eligible for the study if they had adequate clinical and diagnostic data.

The minimum dataset for inclusion in the present study was a detailed medical history collected at the first specialist’s visit, including a structured interview focused on the first clinical signs observed by the parents. The interview included the age of symptoms onset and the following items: suspected vision loss, such as the inability to visually fixate or follow an object or light; visual field abnormality/bumping into objects; inability to recognize colors; worsening of the night vision; increased sensitivity to bright light (i.e. photophobia); and nystagmus. Nystagmus was defined as involuntary, rhythmic, conjugate oscillatory movement of the eyes [17].

In order to confirm the diagnosis of retinal dystrophy we also collected the full report of the initial ophthalmological examination including at the minimum fundus oculi, visual acuity tests appropriate for age and complete electrophysiology. Systemic evaluation and additional non-ophthalmologic assessments (audiometric and vestibular tests, metabolic work-up, kidney function, ultrasounds, neurological examination and brain magnetic resonance imaging) were also searched, to rule out systemic disorders presenting with retinal dystrophy. In order to categorize the degenerative forms, we compared the distribution of signs and symptoms at the first and the last ophthalmological visits, as well as the ERG in at least two time points. Patients who had inadequate data and those with diagnosis of syndromic or metabolic retinal dystrophies were not recruited. The diagnosis of isolated retinal dystrophy was confirmed by retrospective analysis of medical records and electroretinography. The ERG, objectively diagnosed retinal dysfunction even in patients without a visible fundus oculi abnormality. The ERG had specific patterns of retinal dysfunction consistent with the subtype classification of inherited retinal dystrophies.

Patients were categorized into five IRD diagnostic subtypes, according to ophthalmological and ERG criteria [3, 18]: 1) rod-cone dystrophy (RCD) 17 patients (34%); in all the scotopic ERG was severely involved while the photopic ERG had abnormalities of variable severity, deteriorating at follow up.2) cone-rod dystrophy (CRD) 12 (24%); in all the photopic ERG was abnormal while the scotopic ERG was abnormal in five cases, severity of photopic ERG involvement exceeded that of scotopic ERG, at follow up both photopic and scotopic ERGs deteriorated in all cases. 3) achromatopsia (ACHR) 13 (26%); the photopic ERG was severely depressed in all, 3 out of 13 patients had also a mild reduction in amplitude of the scotopic ERG, these findings were stable at follow up. 4) congenital stationary night blindness (CSNB) 6 (12%), all had normal photopic ERG and reduced amplitude of the scotopic ERG. 5) Leber’s congenital amaurosis (LCA) 2 (4%), both patients had severely abnormal ERG early in life.

Analysis included identification of the age of symptoms onset and of the earliest visual signs/symptoms reported by parents, both in the total population and in the five IRD diagnostic subtypes.

Institutional Boards approved the retrospective review of clinical and electrophysiological data.

## Results

In our cohort of 50 children, the symptoms most frequently reported by parents were nystagmus (76%), vision loss (28%) and photophobia (8%). Interestingly, only a minority of parents reported bumping into objects or suspected visual field loss (6%), difficulties with night vision (4%) or problems with color vision (6%). The frequencies of the three most frequent initial symptoms differed among the five IRD subtypes (Table 1).

**Table 1:** Percentage of symptoms and signs reported by children’s parents in the total population and in the five retinal dystrophy subtypes

	Nystagmus	Photophobia	Low vision
Total patients (50 patients)	76%	8%	28%
Rod-cone Distrophy (17 patients)	53%	0%	41%
Cone-rod Distrophy (12 patients)	92%	17%	42%
Achromatopsia (13 patients)	100%	8%	0%
CSNB (6 patients)	67%	0%	19%
Leber’s Amaurosis (2 patients)	50%	50%	100%

LCA = Leber’s congenital amaurosis; ACHR = achromatopsia; CSNB = congenital stationary night blindness; CRD = cone-rod dystrophy; RCD = rod-cone dystrophy

**Fig. 1:**
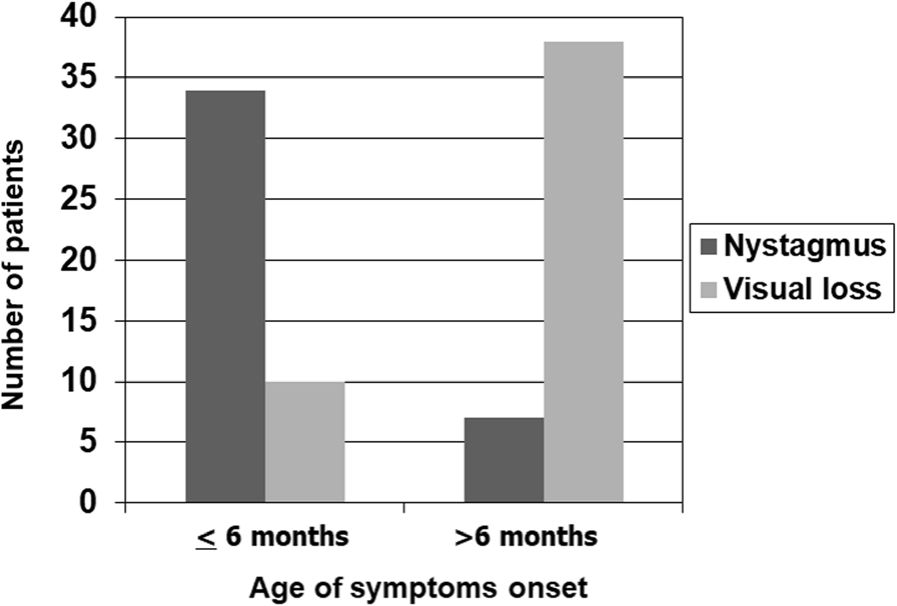
Number of patients with nystagmus and visual loss as symptoms at clinical onset of retinal dystrophy by age groups

The mean age at the symptom onset was 12 months (range 2 months-5 years). The majority of patients (80%) presented before 12 months. Male sex was prevalent in both the group with symptom onset before 12 months and that with symptoms onset after 12 months (57% vs 70% respectively). Nystagmus was the most frequently reported first symptom in children with the onset of symptoms before 6 months of age, while the most frequent symptom presenting after the 6 months of age was visual loss (Fig. 1)

## Discussion

The present study shows that the parents were able to reliably recognize some of the earliest signs of IRD. We identified nystagmus and visual loss as the most constant clinical features reported by parents. The initial clinical symptoms of IRD were apparent before the 12 months of age in the majority of patients of our cohort. Comparing the symptoms by the age of onset, nystagmus was the most frequently reported symptom before the age of six months. By contrast, the onset of abnormal visual behavior suggesting a visual loss, was more frequently reported after 6 months of age.

While nystagmus, particularly the infantile form, can be idiopathic; it can also be a sign of sensory loss due to anterior visual pathways dysfunction, or it can be part of neurological diseases involving the oculomotor system [17, 19]. Clinically, sensory nystagmus worsens by fixing on objects, while neurological nystagmus worsens with eye movements. To distinguish infantile idiopathic nystagmus from sensory defect nystagmus can be challenging. Indeed, about half of the children with infantile nystagmus evaluated by expert pediatric ophthalmologists were subsequently diagnosed with IRDs after electrophysiological evaluation [20]. The etiology of the nystagmus can be confirmed by a combination of electrophysiology, laboratory tests, neurological work up, and imaging [17].

Abnormal visual behavior can represent a significant diagnostic challenge in young children. While this may be a sign of neurological or ophthalmological disorders, in can also represent a delayed visual maturation [21]. Confirming the diagnosis may be particularly difficult in young children, since they are often unable to report sensory loss, or to cooperate with clinical and instrumental testing. Especially in infants, the clinical suspicion relies mostly on parental observation.

When ophthalmic inspection is uninformative, and the diagnostic workup excluded neurological disorders of the visual pathways and global developmental disability, the diagnosis of IRD should be the next to consider. It is noteworthy that the ERG was successfully performed in all children of the present study and, in the appropriate clinical setting, had confirmed the diagnosis in all recruited patients. Electrophysiological techniques are commonly considered difficult to perform in young children and their interpretation requires specific knowledge on normal waveforms at different ages. The ERG reflects the functional activity of retinal photoreceptors, allowing distinction between the cone and rod systems involvement. Different ERG patterns corresponding to selective or prevalent cones or rods dysfunction and their stable or progressive trend during follow up were fundamental for classification of IRD subtypes. This was particularly important for diagnosis when the fundus oculi appearance was normal or non-specific.

Parents of very young children frequently under-report some clinical symptoms of IRD, such as photophobia, color vision, night blindness and peripheral visual field loss. As an example, in IRD forms with cone system involvement, parents appreciated sensitivity to bright light less often than nystagmus and vision loss, even in achromatopsia where photophobia is an early and distinctive feature. Currently there are no pediatric reports describing pathognomonic clinical features that may constitute red flags to alert clinicians to suspect the diagnosis of IRD. In adults, recognizing the patterns of initial symptoms allowed the differentiation between the cone dystrophies, with early severe visual loss, from earlier rods dysfunction, such as retinitis pigmentosa with the initial symptom of night blindness [7].

Our study shows that in pediatric population, the early symptoms of IRD reported by parents have less defined patterns, as compared to the adult patients with IRD [7–13]. We hypothesize that there are two main reasons for this. The first reason is a parental under-reporting of symptoms that are difficult to recognize in very young children. The second reason is the more aggressive and more diffuse nature of retinal pathology in the early onset IRDs, leading to the involvement of both, rod and cone systems early in the course of disease.

What this study adds is the delineation of the early clinical features in children with IRD, observable by the parents, which could in turn help the pediatricians and other primary care providers to suspect the disease.

Based on the results of our study we suggest that comprehensive evaluation of a child seen for a complaint of vision loss or nystagmus, should include detailed questions looking for specific signs and symptoms of rods and cones dysfunction, both often overlooked and underreported. Presence of any of the symptoms should alert the pediatrician to consider IRDs in the differential diagnosis of vision loss and nystagmus.

A limitation of the present study was a relatively small number of recruited subjects, precluding more detailed analysis of clinical characteristics and age at onset by retinal subgroups, as well as good statistical analysis. Even if the focus of our study was to describe the early clinical clues that would increase the pediatrician’s suspicion for retinal disorders, and we used electroretinography to confirm the diagnosis, the lack of genetic data is a limitation of the present study. Nowadays significant achievements have been made in the elucidation of the genetic causes of many IRDs [22] and to perform genetic diagnosis should be be the final step of the diagnostic pathway. Molecular studies are currently under way in many of our patients and will be available in the near future for further analysis.

## Conclusions

In conclusion, the results of our study emphasize the importance of prompt ophthalmological evaluation of infants and children presenting with nystagmus and/or suspected vision loss. To suspect IRDs, medical history should specifically focus on the presence of light sensitivity and night blindness. Abnormalities of the visual fields and color vision should be documented whenever possible. It is important to recognize that parents might not readily recognize and report these symptoms. The timely identification of early clinical clues suggesting pediatric IRDs, will expedite the diagnostic process and therefore allowing the affected children to greatly benefit from early therapies and treatments which can have very important implication for children’s quality of life.

## Data Availability

S.Anna University Hospital of Ferrara, study-room of Prof. Suppiej.
